# Supplementary material for: A Humanized Yeast Phenomic Model of Deoxycytidine Kinase to Predict Genetic Buffering of Nucleoside Analog Cytotoxicity
Source: Genes (Basel). 2019 Sep 30;10(10):770. doi: 10.3390/genes10100770 (PMC6826991; doi:10.3390/genes10100770)
Supplement: Supplementary file 1 [file genes-10-00770-s001.zip › Additional_Files/Additional_File7_GOTermHeatmaps.zip!/Additional_File7_GOTermHeatmaps/enzyme_activator_activity.pdf]

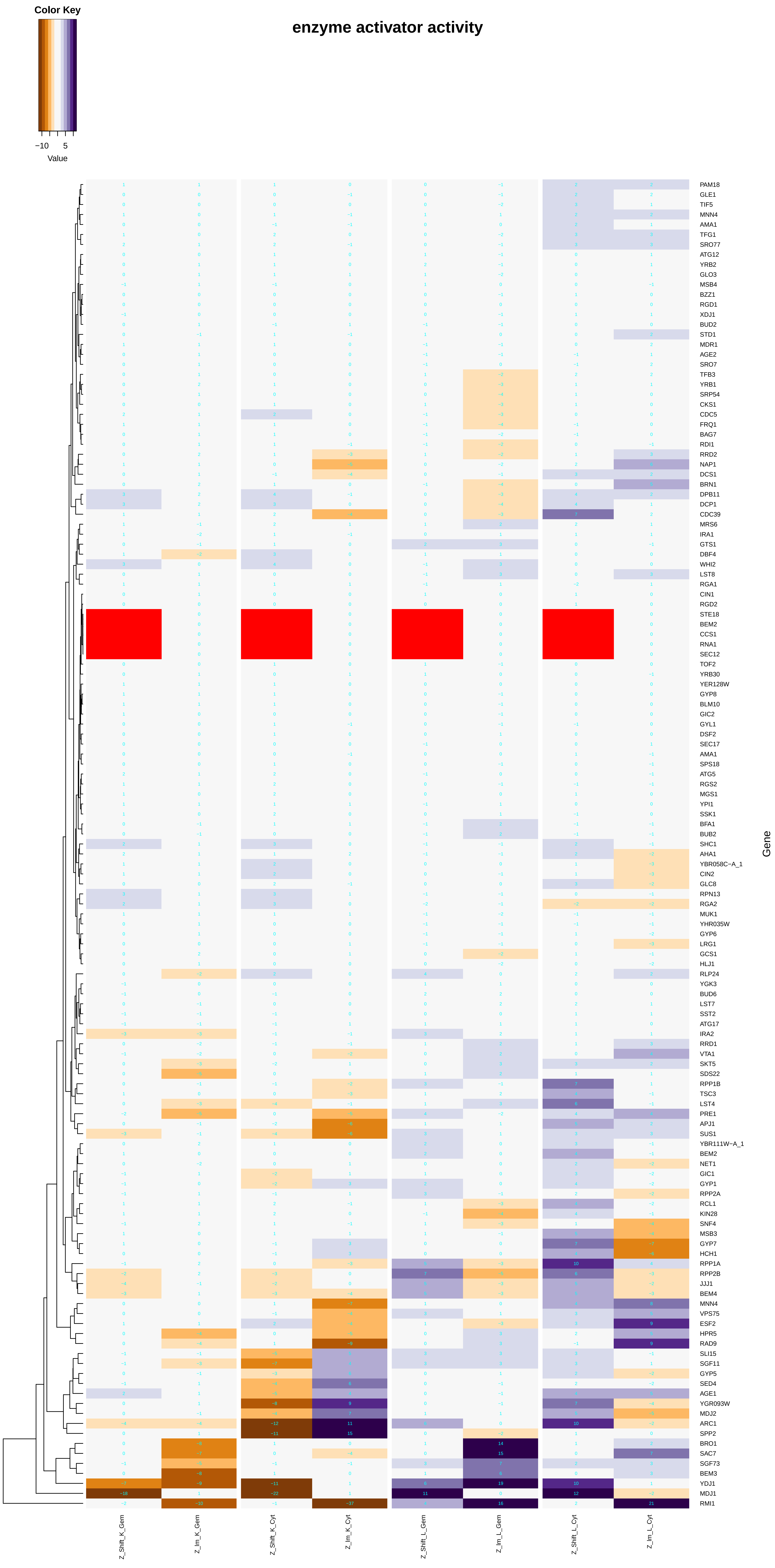

enzyme activator activity
Color Key
−10
5
Value
PAM18
GLE1
TIF5
MNN4
AMA1
TFG1
SRO77
ATG12
YRB2
GLO3
MSB4
BZZ1
RGD1
XDJ1
BUD2
STD1
MDR1
AGE2
SRO7
TFB3
YRB1
SRP54
CKS1
CDC5
FRQ1
BAG7
RDI1
RRD2
NAP1
DCS1
BRN1
DPB11
DCP1
CDC39
MRS6
IRA1
GTS1
DBF4
WHI2
LST8
RGA1
CIN1
RGD2
STE18
BEM2
CCS1
RNA1
SEC12
TOF2
YRB30
YER128W
GYP8
BLM10
GIC2
GYL1
DSF2
SEC17
AMA1
SPS18
ATG5
RGS2
MGS1
YPI1
SSK1
BFA1
BUB2
SHC1
AHA1
YBR058C−A_1
CIN2
GLC8
RPN13
RGA2
MUK1
YHR035W
GYP6
LRG1
GCS1
HLJ1
RLP24
YGK3
BUD6
LST7
SST2
ATG17
IRA2
RRD1
VTA1
SKT5
SDS22
RPP1B
TSC3
LST4
PRE1
APJ1
SUS1
YBR111W−A_1
BEM2
NET1
GIC1
GYP1
RPP2A
RCL1
KIN28
SNF4
MSB3
GYP7
HCH1
RPP1A
RPP2B
JJJ1
BEM4
MNN4
VPS75
ESF2
HPR5
RAD9
SLI15
SGF11
GYP5
SED4
AGE1
YGR093W
MDJ2
ARC1
SPP2
BRO1
SAC7
SGF73
BEM3
YDJ1
MDJ1
RMI1
Gene
Z_Shift_K_Gem
Z_Im_K_Gem
Z_Shift_K_Cyt
Z_Im_K_Cyt
Z_Shift_L_Gem
Z_Im_L_Gem
Z_Shift_L_Cyt
Z_Im_L_Cyt

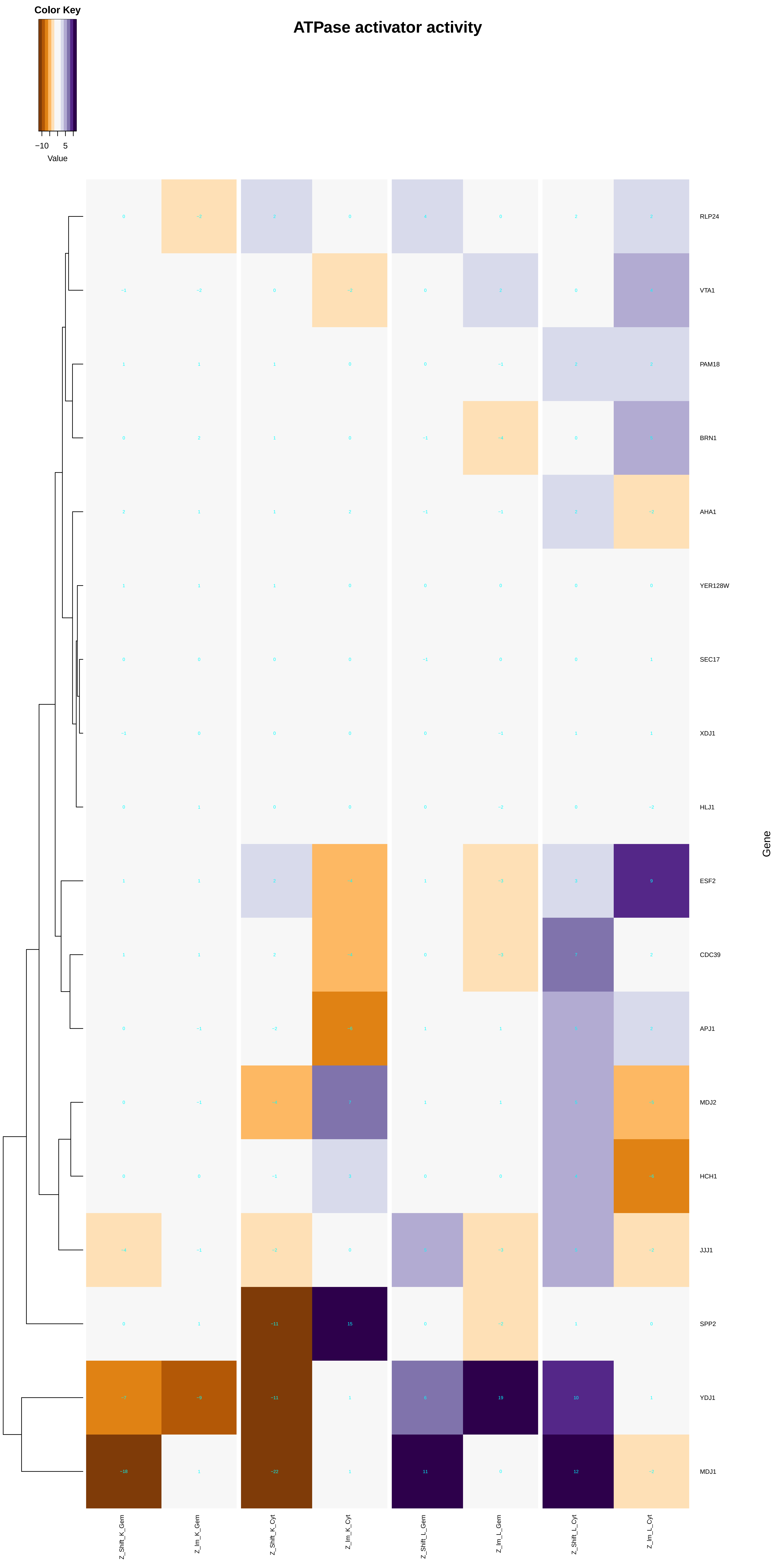
ATPase activator activity
Color Key
−10
5
Value
RLP24
VTA1
PAM18
BRN1
AHA1
YER128W
SEC17
XDJ1
HLJ1
ESF2
CDC39
APJ1
MDJ2
HCH1
JJJ1
SPP2
YDJ1
MDJ1
Gene
Z_Shift_K_Gem
Z_Im_K_Gem
Z_Shift_K_Cyt
Z_Im_K_Cyt
Z_Shift_L_Gem
Z_Im_L_Gem
Z_Shift_L_Cyt
Z_Im_L_Cyt

GTPase activator activity

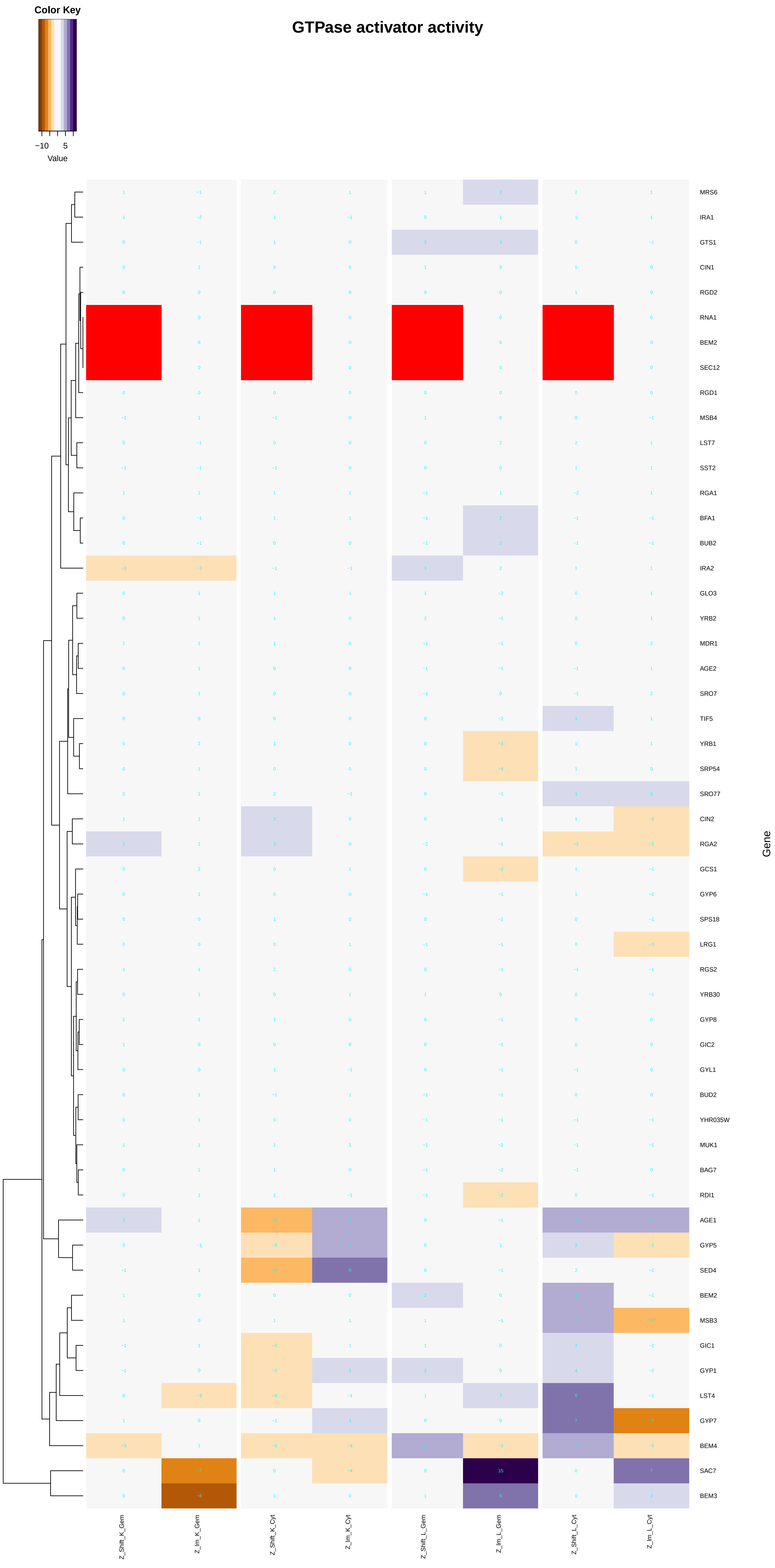

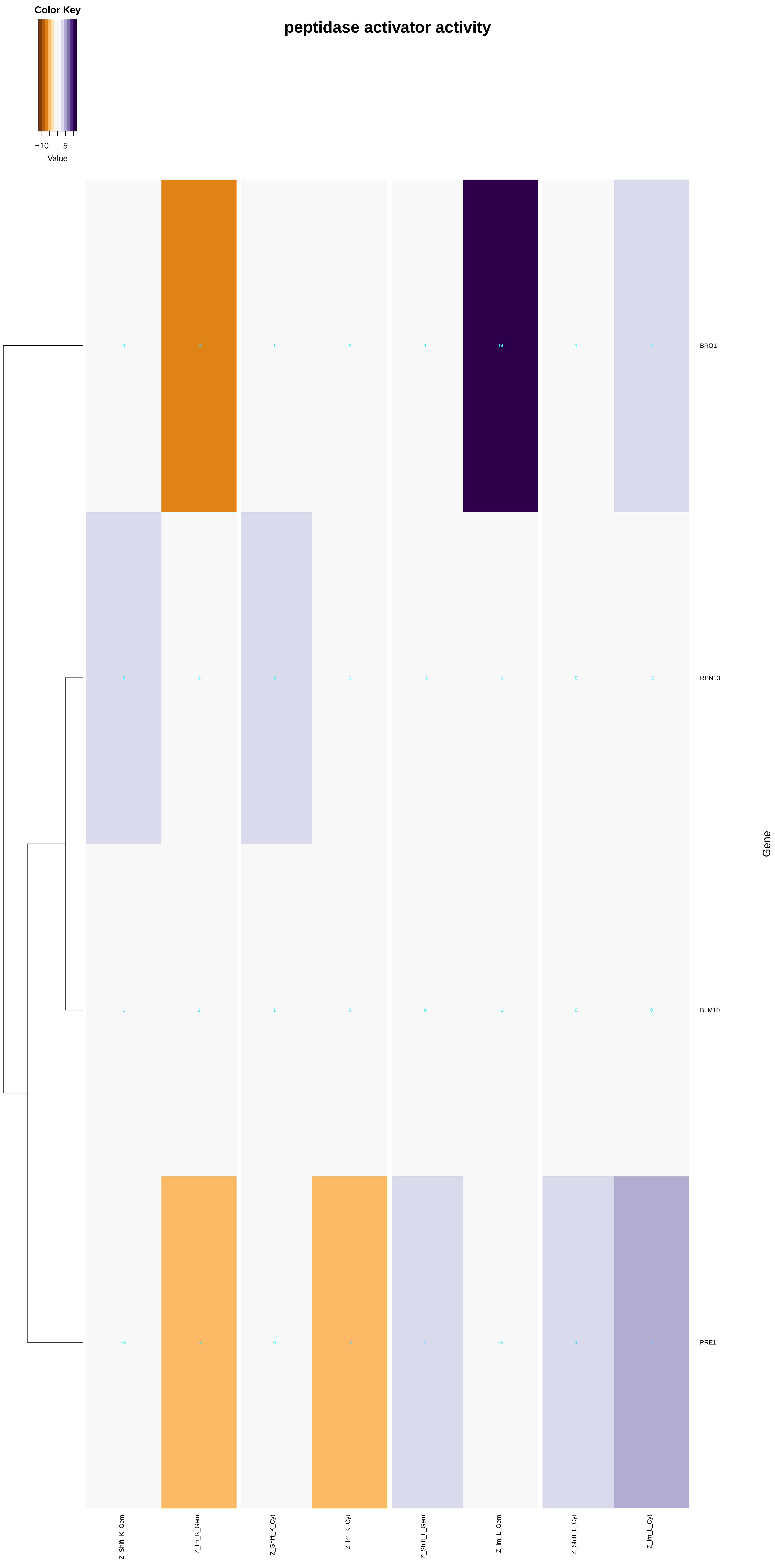

Color Key
−10
5
Value
peptidase activator activity
BRO1
RPN13
BLM10
PRE1
Gene
Z_Shift_K_Gem
Z_Im_K_Gem
Z_Shift_K_Cyt
Z_Im_K_Cyt
Z_Shift_L_Gem
Z_Im_L_Gem
Z_Shift_L_Cyt
Z_Im_L_Cyt

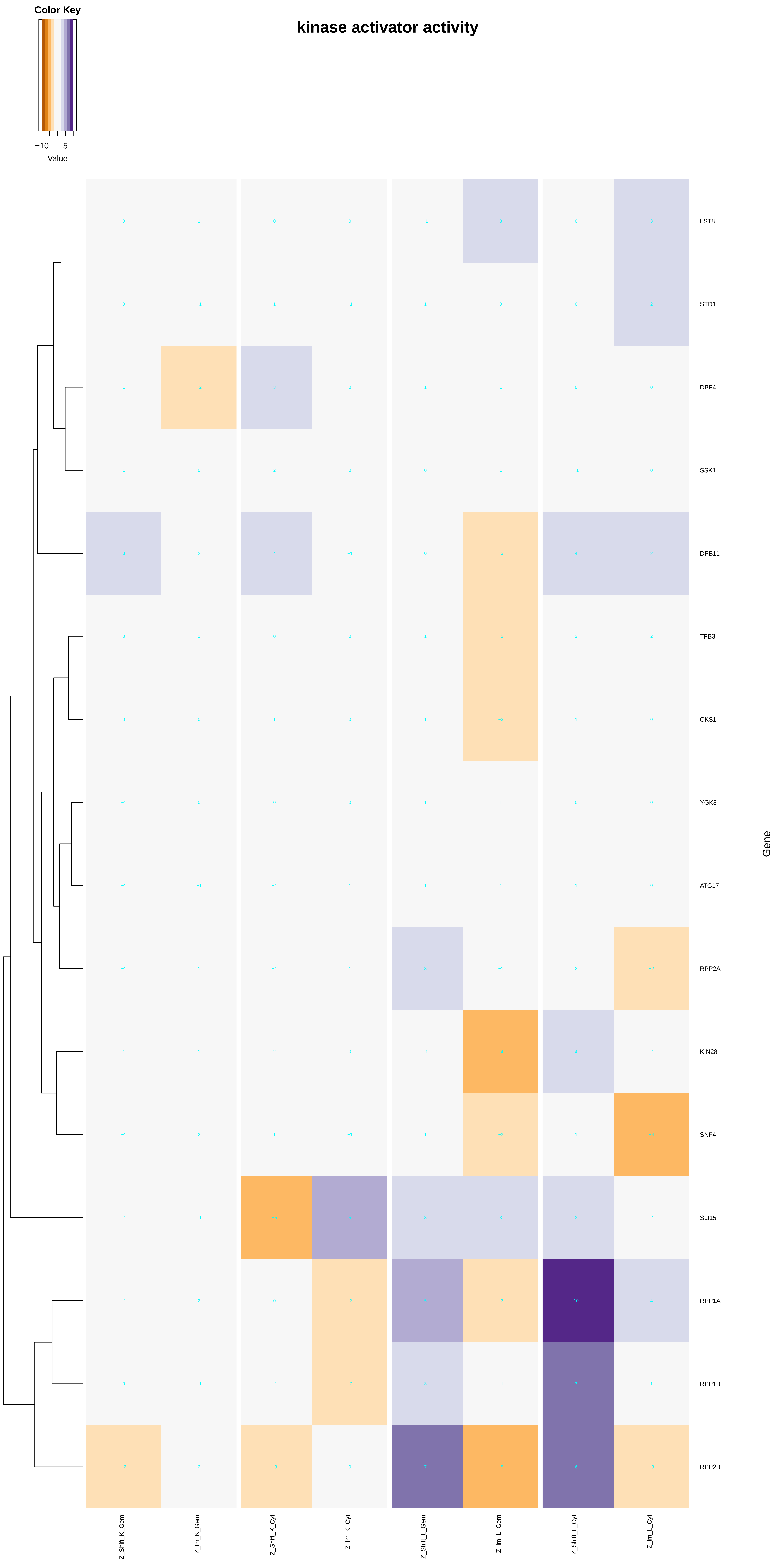
kinase activator activity
Color Key
−10
5
Value
LST8
STD1
DBF4
SSK1
DPB11
TFB3
CKS1
YGK3
ATG17
RPP2A
KIN28
SNF4
SLI15
RPP1A
RPP1B
RPP2B
Gene
Z_Shift_K_Gem
Z_lm_K_Gem
Z_Shift_K_Cyt
Z_lm_K_Cyt
Z_Shift_L_Gem
Z_lm_L_Gem
Z_Shift_L_Cyt
Z_lm_L_Cyt

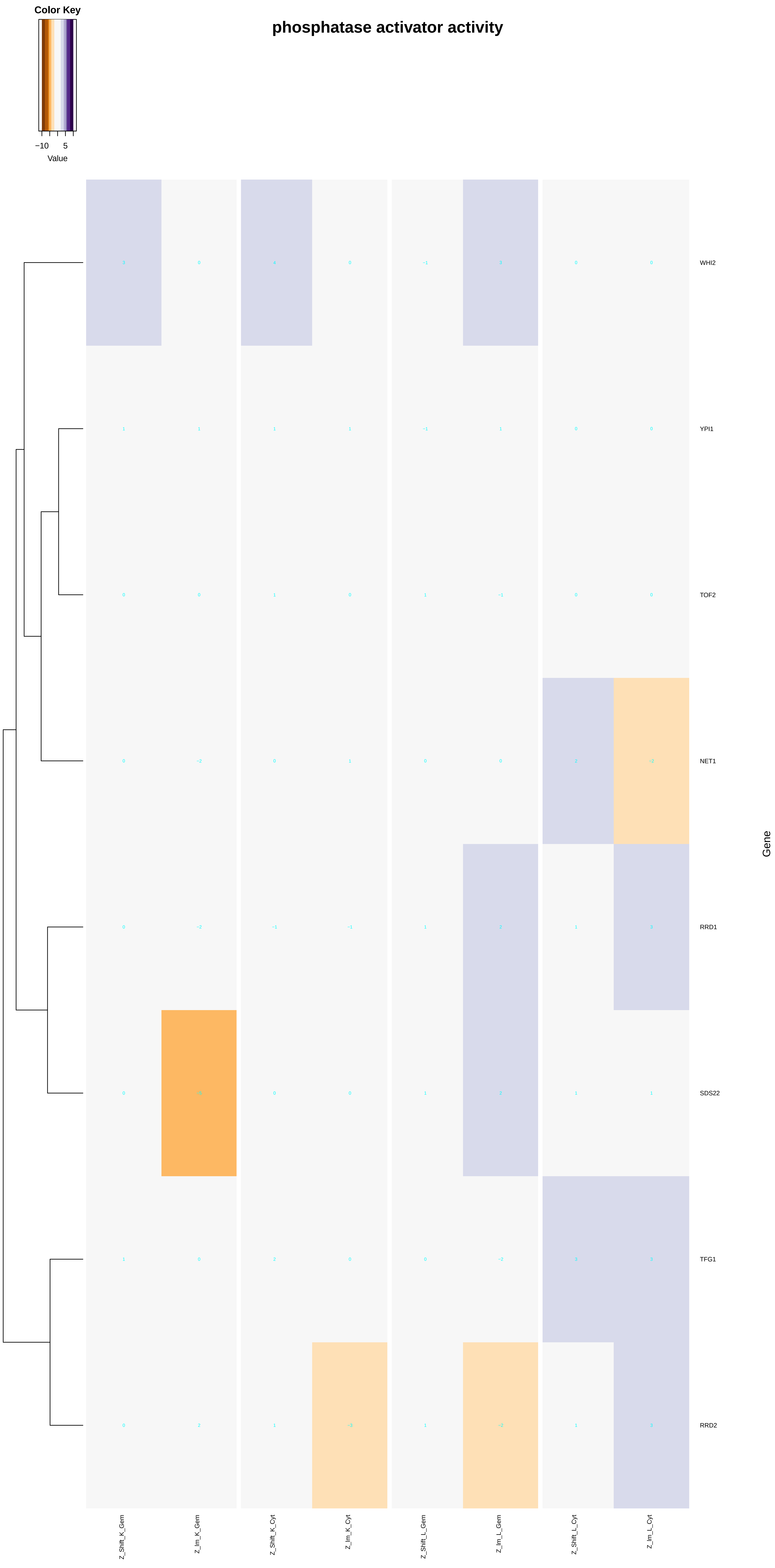
phosphatase activator activity
Color Key
−10
5
Value
WHI2
YPI1
TOF2
NET1
RRD1
SDS22
TFG1
RRD2
Gene
Z_Shift_K_Gem
Z_Im_K_Gem
Z_Shift_K_Cyt
Z_Im_K_Cyt
Z_Shift_L_Gem
Z_Im_L_Gem
Z_Shift_L_Cyt
Z_Im_L_Cyt

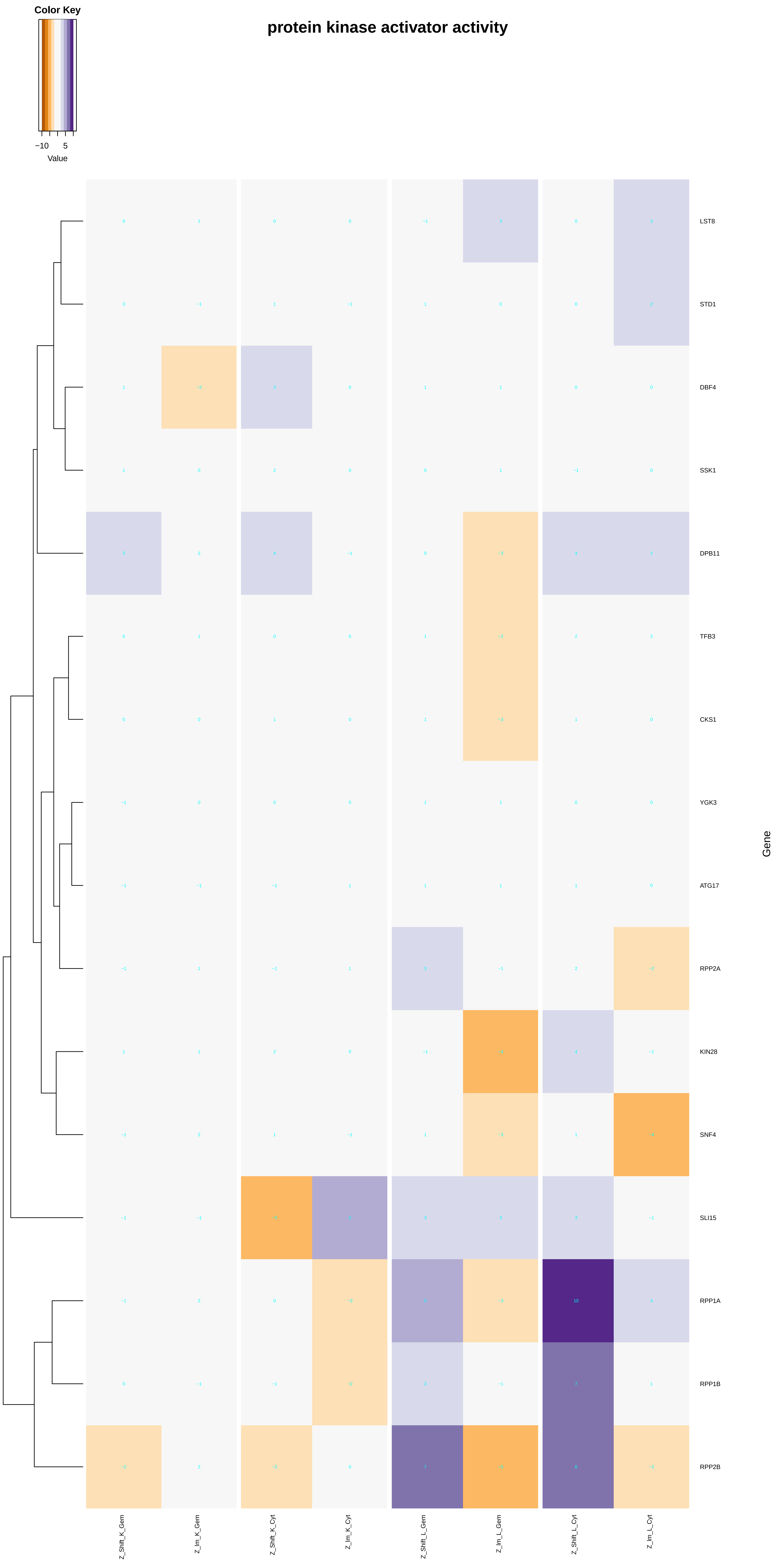
Color Key
−10
5
Value
protein kinase activator activity
LST8
STD1
DBF4
SSK1
DPB11
TFB3
CKS1
YGK3
ATG17
RPP2A
KIN28
SNF4
SLI15
RPP1A
RPP1B
RPP2B
Gene
Z_Shift_K_Gem
Z_Im_K_Gem
Z_Shift_K_Cyt
Z_Im_K_Cyt
Z_Shift_L_Gem
Z_Im_L_Gem
Z_Shift_L_Cyt
Z_Im_L_Cyt

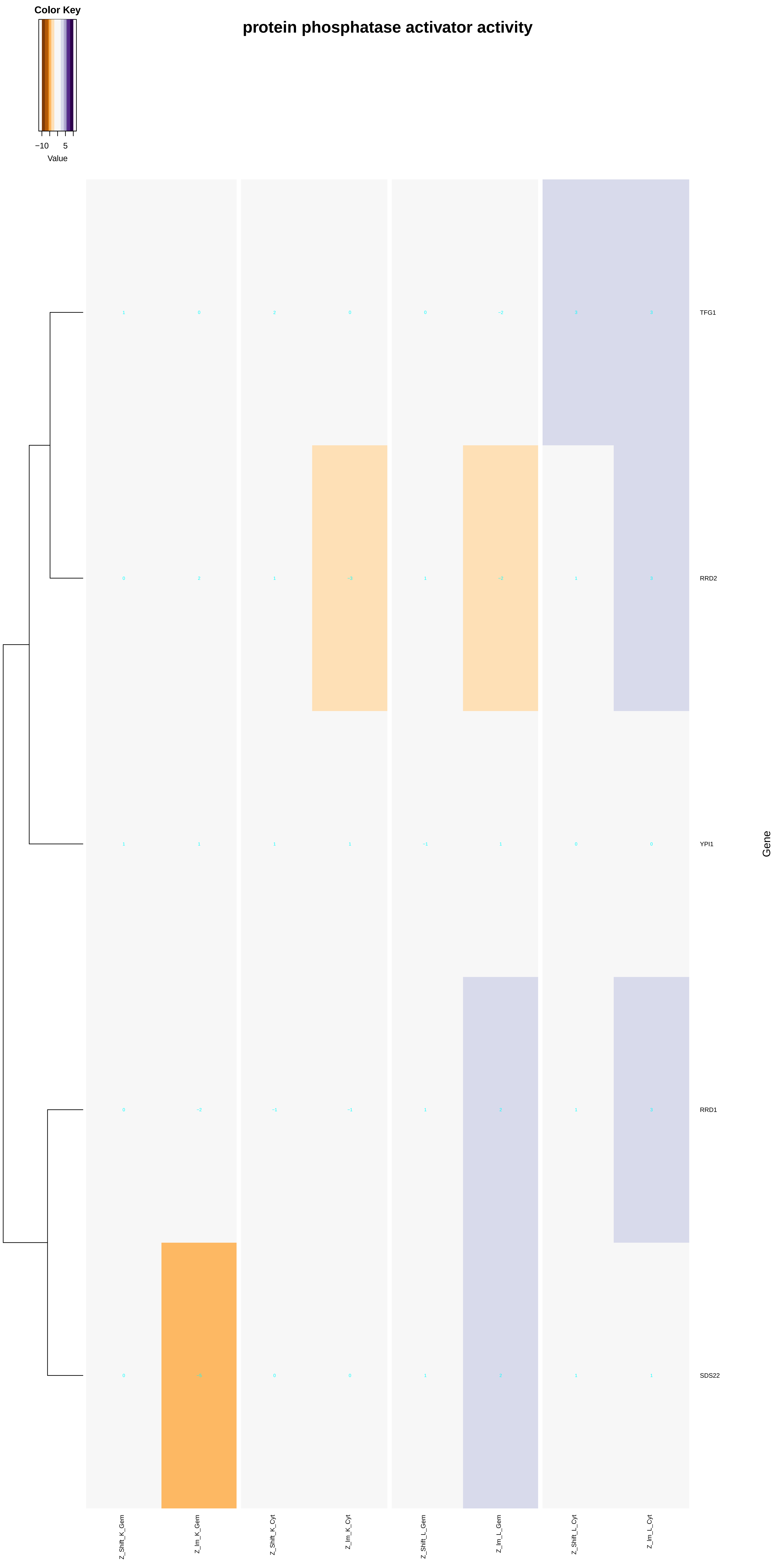

Color Key
−10
5
Value
protein phosphatase activator activity
TFG1
RRD2
YPI1
RRD1
SDS22
Gene
Z_Shift_K_Gem
Z_Im_K_Gem
Z_Shift_K_Cyt
Z_Im_K_Cyt
Z_Shift_L_Gem
Z_Im_L_Gem
Z_Shift_L_Cyt
Z_Im_L_Cyt

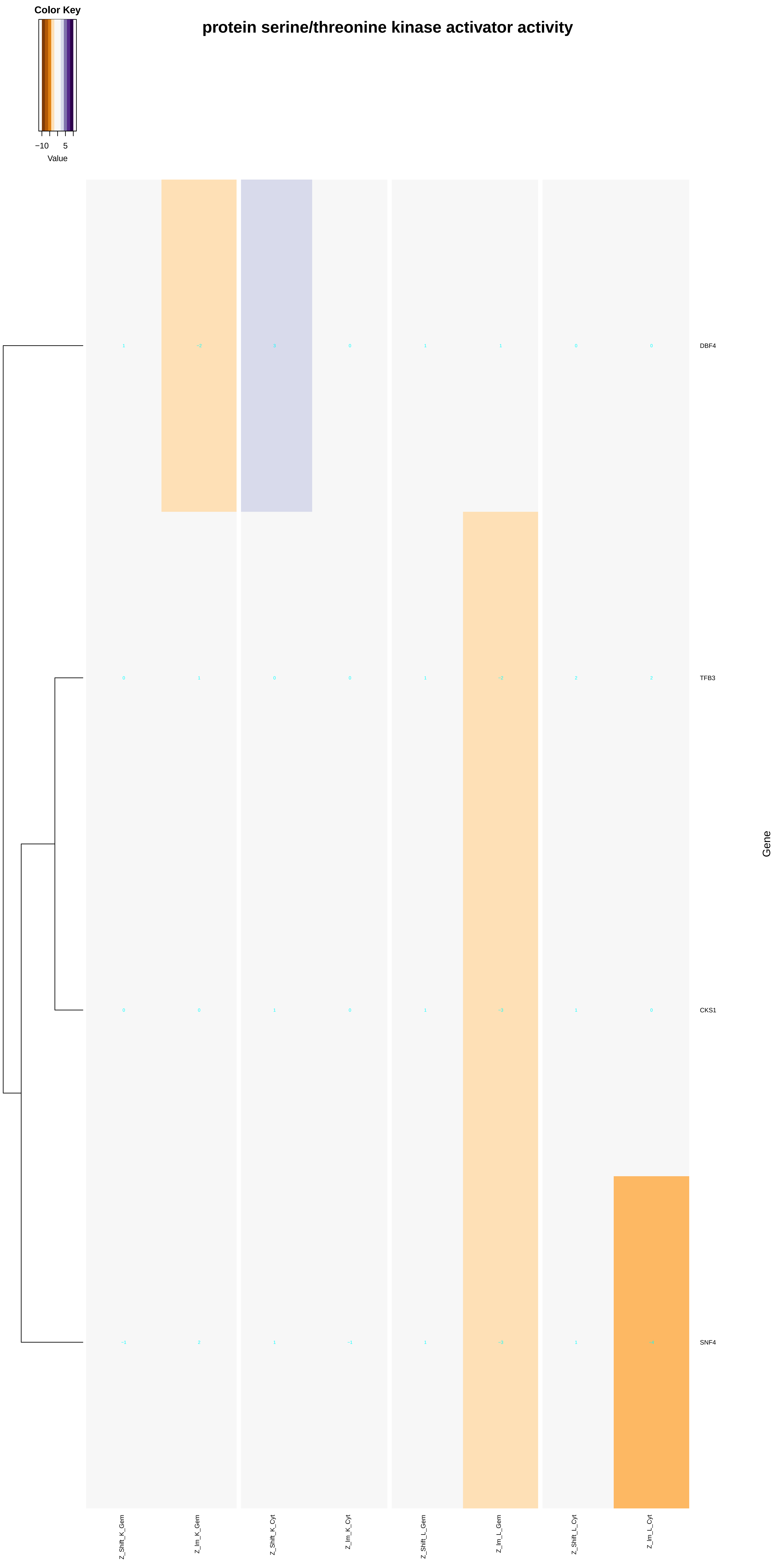
protein serine/threonine kinase activator activity
Color Key
−10
5
Value
DBF4
TFB3
CKS1
SNF4
Gene
Z_Shift_K_Gem
Z_Im_K_Gem
Z_Shift_K_Cyt
Z_Im_K_Cyt
Z_Shift_L_Gem
Z_Im_L_Gem
Z_Shift_L_Cyt
Z_Im_L_Cyt
